# Phos-tag analysis of Rab10 phosphorylation by LRRK2: a powerful assay for assessing kinase function and inhibitors

**DOI:** 10.1042/BCJ20160557

**Published:** 2016-08-30

**Authors:** Genta Ito, Kristina Katsemonova, Francesca Tonelli, Pawel Lis, Marco A.S. Baptista, Natalia Shpiro, Graham Duddy, Steve Wilson, Philip Wing-Lok Ho, Shu-Leong Ho, Alastair D. Reith, Dario R. Alessi

**Affiliations:** * MRC Protein Phosphorylation and Ubiquitylation Unit, School of Life Sciences, University of Dundee, Dundee DD1 5EH, U.K.; † The Michael J. Fox Foundation for Parkinson's Research, Grand Central Station, P.O. Box 4777, New York, NY 10163, U.S.A.; ‡ Molecular Discovery Research, GlaxoSmithKline Pharmaceuticals R&D, New Frontiers Science Park, Harlow, Essex CM19 5AD, U.K.; § RD Platform Technology & Science, GlaxoSmithKline, U.K.; ║ Division of Neurology, Department of Medicine, University of Hong Kong, Hong Kong; ¶ Neurodegeneration Discovery Performance Unit, RD Neurosciences, GlaxoSmithKline Pharmaceuticals R&D, Stevenage, U.K.

**Keywords:** Parkinson's disease, protein kinases, Rab GTPase, signal transduction

## Abstract

Autosomal dominant mutations that activate the leucine-rich repeat kinase 2 (LRRK2) cause inherited Parkinson's disease. Recent work has revealed that LRRK2 directly phosphorylates a conserved threonine/serine residue in the effector-binding switch-II motif of a number of Rab GTPase proteins, including Rab10. Here we describe a facile and robust method to assess phosphorylation of endogenous Rab10 in mouse embryonic fibroblasts (MEFs), lung and spleen-derived B-cells, based on the ability of the Phos-tag reagent to retard the electrophoretic mobility of LRRK2-phosphorylated Rab10. We exploit this assay to show that phosphorylation of Rab10 is ablated in kinase-inactive LRRK2[D2017A] knockin MEFs and mouse lung, demonstrating that LRRK2 is the major Rab10 kinase in these cells/tissue. We also establish that the Phos-tag assay can be deployed to monitor the impact that activating LRRK2 pathogenic (G2019S and R1441G) knockin mutations have on stimulating Rab10 phosphorylation. We show that upon addition of LRRK2 inhibitors, Rab10 is dephosphorylated within 1–2 min, markedly more rapidly than the Ser^935^ and Ser^1292^ biomarker sites that require 40–80 min. Furthermore, we find that phosphorylation of Rab10 is suppressed in LRRK2[S910A+S935A] knockin MEFs indicating that phosphorylation of Ser^910^ and Ser^935^ and potentially 14-3-3 binding play a role in facilitating the phosphorylation of Rab10 by LRRK2 *in vivo*. The Rab Phos-tag assay has the potential to significantly aid with evaluating the effect that inhibitors, mutations and other factors have on the LRRK2 signalling pathway.

## INTRODUCTION

Our knowledge of the origins of Parkinson's disease has been transformed by the identification of genes whose mutation in humans leads to Mendelian inherited disease [1,2]. One of these genes encodes the leucine-rich repeat kinase 2 (LRRK2) protein kinase where autosomal dominant mutations account for ∼1% of sporadic Parkinson's disease [3,4]. The most common LRRK2 mutation converts Gly^2019^ into a serine within the kinase domain magnesium ion-binding motif [5]. This mutation enhances *in vitro* protein kinase activity ∼3-fold [6,7], indicating that abnormal increase in the kinase activity of LRRK2 is involved in the pathogenesis of Parkinson's disease, suggesting that LRRK2 kinase inhibitors have therapeutic benefit for the treatment of Parkinson's disease. LRRK2 is a large enzyme (2527 residues), consisting of leucine-rich repeats (residues 1010–1287), a GTPase domain (residues 1335–1504), a COR [C-terminal of ROC (Ras of complex GTPase domain)] domain (residues 1517–1843), a serine/threonine protein kinase domain (residues 1875–2132) and a WD40 repeat (residues 2231–2276) [8]. Three well-characterized pathogenic mutations occur within the GTPase domain (R1441C, R1441G and R1441H) [9,10] and one within the COR domain (Y1699C) [11]. Unlike the G2019S mutation, the R1441G/H/C and Y1699C mutations do not directly enhance LRRK2 *in vitro* kinase activity [12].

We recently reported that members of the Rab GTPase family, including Rab8A and Rab10 were direct physiological substrates for LRRK2 [13]. The LRRK2 phosphorylation site (Thr^72^ for Rab8A and Thr^73^ for Rab10) is conserved in ∼50 different Rab proteins [13], and lies within the effector-binding switch-II motif [14–16]. LRRK2 phosphorylation of Rab8A and Rab10 proteins is inhibitory as it suppresses binding to the Rab GDP-dissociation inhibitor (GDI) factors that are required for membrane delivery and recycling [13]. Furthermore, LRRK2 phosphorylation also inhibits binding of Rab8A to Rabin-8, its guanine-nucleotide-exchange factor (GEF) activator [13].

Other work has also linked Rab GTPases with Parkinson's disease. For example, Rab7L1 (also known as Rab29) is one of five genes that is mutated with Parkinson's disease patients that have the PARK16 mutation [17,18]. Depletion of Rab7L1 reportedly induced loss of dopaminergic neurons, similar to that observed with LRRK2-[G2019S] expression [19]. Furthermore, genetic analysis has recently revealed that loss of function mutations in the poorly studied Rab39B protein is responsible for a rare form of X-linked Parkinson's disease [20,21]. Moreover, overexpression of Rab8a, Rab1 and Rab3a protein attenuated α-synuclein-induced cytotoxicity in cellular and animal models of Parkinson's disease [22,23]. Finally, another protein kinase mutated in Parkinson's disease termed PINK1, indirectly controls the phosphorylation of a small group of Rabs including Rab8A at a site distinct from that used by LRRK2 (Ser^111^ on Rab8A) [24]. Taken together these results strongly suggest a functional interplay between Rab GTPases and known Parkinson's disease factors.

In 2004, an agent (1,3-bis[bis(pyridin-2-ylmethyl) amino]propan-2-olato dizinc(II) complex) commonly referred to as ‘Phos-tag’ was described that binds to phosphate ions with much higher affinity (*K*_d_ ∼ 25 nM for phenyl phosphate) than other anions [25]. The Phos-tag reagent was subsequently shown to interact with high affinity with proteins as well as peptides phosphorylated on serine, threonine and tyrosine residues [26]. A modified version of the Phos-tag reagent termed ‘Phos-tag acrylamide’ (*N*-(5-(2-acryloylaminoethylcarbamoyl)pyridin-2-ylmethyl)-*N*,*N*′,*N*′-tris(pyridin-2-yl-methyl)-1,3-diaminopropan-2-ol) was developed that when polymerized into SDS/polyacrylamide gels retarded electrophoretic mobility of phosphorylated proteins, resulting in substantial mobility shifts [27]. The Phos-tag approach is particularly suited for analysing phosphorylation of relatively small proteins such as Rab protein that are phosphorylated at a single site. We previously observed that in a human embryonic kidney (HEK)-293 cell overexpression system, LRRK2 phosphorylation of haemagglutinin (HA)–Rab8A and HA–Rab10 resulted in an electrophoretic mobility shift of the phosphorylated Rab protein [13]. We also observed that pathogenic LRRK2 mutations tested, including the R1441G, Y1699C and G2019S, stimulated phosphorylation of Rab protein to a greater extent than wild-type (WT) LRRK2 [13].

An important goal is to develop robust methods to rapidly assess LRRK2 phosphorylation of endogenous Rab proteins in samples where sample material may be limiting. In the present study we develop a straightforward procedure based on the Phos-tag approach to quantitatively assess phosphorylation of endogenous Rab10 in mouse embryonic fibroblasts (MEFs), lung tissue as well as spleen-derived B-cells. We demonstrate that ablation of LRRK2 catalytic activity in a novel kinase-inactive LRRK2[D2017A] knockin mouse model blocks Rab10 phosphorylation in MEFs as well as lung, demonstrating that LRRK2 is indeed the major Rab10 kinase in these cells and tissue. We establish that the Phos-tag assay can be used to monitor the impact of LRRK2 inhibitors, as well as pathogenic knockin mutations (G2019S and R1441G) on Rab10 phosphorylation. There is also significant interest in studying the roles that LRRK2 Ser^910^ and Ser^935^ phosphorylation play, as phosphorylation of these residues promotes 14-3-3 binding and LRRK2 inhibitors induce their dephosphorylation [12,28]. To address whether Ser^910^ and Ser^935^ play a role in regulating Rab10 phosphorylation *in vivo*, we generated LRRK2[S910A+S935A] knockin MEFs and found that this mutation significantly inhibits Rab10 phosphorylation. The Rab10 Phos-tag assay will aid assessment of the impact that inhibitors, mutations and other factors have on the LRRK2 signalling pathway.

## MATERIALS AND METHODS

### Reagents

GSK2578215A was obtained from GlaxoSmithKline [29]. HG-10-102-01 was custom synthesized as described previously [30]. MLi-2 was obtained from Merck and also synthesized as described in [31a]. Phos-tag acrylamide was synthesized as described in [31b]. Phos-tag acrylamide was stored at 5 mM aqueous solution (3.43 mg of compound in 1 ml of solution) at 4°C in black tubes that block out light as Phos-tag acrylamide is light-sensitive. HPLC analysis of stock Phos-tag acrylamide was undertaken every 4–5 weeks to ensure stock had not started to polymerize. All recombinant proteins, DNA constructs and antibodies generated for the present study can be requested via our reagents website (https://mrcppureagents.dundee.ac.uk/).

### General methods

DNA procedures were undertaken using standard protocols. DNA constructs were purified from *E. coli* DH5α using a Maxi Prep kit (Qiagen). DNA sequence of the DNA constructs used in the present study was performed by our Sequencing Service (http://www.dnaseq.co.uk).

### Antibodies

Anti-Rab10 antibody was from Cell Signaling Technology (#8127) and used at 1:1000 dilution. Rabbit monoclonal antibodies for total LRRK2 (UDD3) and pS935-LRRK2 (UDD2) were purified at the University of Dundee and used at 1:10000 and 1:2000 dilutions respectively. Rabbit monoclonal antibody detecting phospho-Ser^1292^ LRRK2 was from Abcam (ab203181) and used at a final concentration of 1 μg/ml. Anti-glyceraldehyde-3-phosphate dehydrogenase (GAPDH) antibody was from Santa Cruz Biotechnology (sc-32233) and used at 1:5000 dilution. Sheep polyclonal antibody for phospho-Thr^73^ Rab10 (S873D) was described previously [13] and used at final concentration of 1 μg/ml in the presence of 10 μg/ml non-phosphorylated peptide. Horseradish peroxidase-conjugated anti-mouse (#31450), -rabbit (#31460), -rat (#31470) and -sheep IgG secondary antibodies (#31480) were from Thermo Fisher Scientific.

### Plasmids

The following constructs were used for protein production: 6His-SUMO-Rab10 WT (DU51062), 6His-SUMO-Rab8A WT (DU47363). The following constructs were used for overexpression in cells: HA–Rab10 WT/T73A (DU44250/DU51006), FLAG–LRRK2 R1441G (DU13077). The following constructs were used for generation of Rab10 knockout (KO) A549 cells: Rab10 KO N-terminal antisense guide and Cas9 D10A (DU52110) and Rab10 KO N-terminal sense guide (DU52100). Full datasheets for each plasmid are available from https://mrcppureagents.dundee.ac.uk/.

### Mice

All animal studies were ethically reviewed and carried out in accordance with Animals (Scientific Procedures) Act 1986, the GSK Policy on the Care, Welfare and Treatment of Animals, regulations set by the University of Dundee and the U.K. Home Office. Animal studies and breeding were approved by the University of Dundee ethical committee and performed under a U.K. Home Office project licence and maintained under specific pathogen-free conditions at the University of Dundee. Animals (unless otherwise stated) were multiply housed at an ambient temperature (20–24°C) and humidity (45–55%) maintained on a 12 h light/12 h dark cycle, with free access to food (SDS RM No. 3 autoclavable) and water.

The LRRK2[G2019S]^GSK^ knockin mice, the LRRK2[A2016T] knockin mice and the LRRK2[R1441G] knockin mice were described previously [13,32]. The LRRK2 KO mice were generated and provided by Dr Huaibin Cai (National Institutes of Health, Bethesda, MD, U.S.A.) and have been described previously [33].

For experiments shown in Figures 5(B) and 7, littermate matched WT and LRRK2 knockin mice (3–6 months of age) were injected subcutaneously either with vehicle [40% (w/v) (2-hydroxypropyl)-β-cyclodextrin (Sigma–Aldrich)] or MLi-2 dissolved in vehicle at the indicated dose and killed by cervical dislocation 1 h after treatment. Lung was rapidly isolated and snap frozen in liquid nitrogen. No specific randomization method or blinding was applied to experiments.

### Generation of LRRK2[D2017A] knockin mice

The LRRK2[D2017A] knockin mouse line was generated by a targeting strategy devised to introduce the point mutation D2017A into exon 41 of the *LRRK2* gene by homologous recombination in mouse embryonic stem (ES) cells. 5′ and 3′ homology arms (approximately 4.8 and 3.8 kb respectively) flanking exon 41 were generated using Phusion High-Fidelity DNA Polymerase (New England Biolabs) on a C57BL/6J genomic DNA template. Similarly, a 739 bp fragment carrying exon 41 lying between these two homology arms was isolated and subjected to site-directed mutagenesis with the QuikChangeII site-directed mutagenesis kit (Stratagene) to introduce the appropriate point mutation (A to C mutation at bp 102 of exon 41). The 5′ and 3′ homology arms and the mutated exon 41 fragments were subcloned into a parental targeting vector to achieve the positioning of the loxP and FRT sites and PGKneo cassette. Gene targeting was performed in *de novo* generated hybrid C57BL/6J;129Ola-derived ES cells. The targeting construct was linearized and electroporated into ES cells according to standard methods. ES cells correctly targeted at the 3′ end was identified by Southern blot analysis of EcoRV digested genomic DNA using a PCR-derived external probe. Correct gene targeting at the 5′ end and presence of the point mutation was confirmed by sequencing of a ∼6 kb PCR product. The latter was generated by high-fidelity PCR of ES cell clone-derived genomic DNA using primers spanning the 5′ homology arm. Correctly targeted ES cell clones were injected into BALB/c blastocysts and implanted into foster mothers according to standard procedures. Male chimaeras resulting from the D2017A-targeted ES cells were bred with C57BL/6J female mice expressing CRE recombinase from the ROSA26 locus to facilitate removal of the loxP flanked PGKneo cassette *in vivo*, and germline transmission of the targeted allele was confirmed by PCR. Germline mice were back-crossed once to C57BL/6J mice, and confirmed to be >98% C57BL/6J by single nucleotide polymorphism (SNP) analysis. The line was subsequently maintained by breeding with C57BL/6J, and crossing mice heterozygous for the point mutation generated homozygous mice at the expected Mendelian ratio. Separate colonies of WT and homozygous animals were subsequently maintained to minimize breeding wastage. Standard genotyping which distinguishes WT from point mutation knockin alleles was used throughout. Genotyping of mice was performed by PCR using genomic DNA isolated from ear biopsies.

### Generation of LRRK2[S910A+S935A] knockin mice

The constitutive LRRK2[S910A+S935A] knockin mouse line was produced by implementing a targeting strategy based on NCBI transcript NM_025730.3, to introduce two point mutations S910A and S935A into exon 21 of the *LRRK2* gene by homologous recombination in mouse ES cells (TaconicArtemis). To start with, the S910A and S935A mutations have been introduced into exon 21 by site-directed mutagenesis with the QuikChangeII site-directed mutagenesis kit (Stratagene) (S910A: TCA to GCC and S935A: TCG to GCG of exon 21). The positive selection marker PuroR has been flanked by FRT sites and inserted into intron 21. 5′ and 3′ homology arms (approximately 4.1 and 6 kb respectively) flanking exon 21 were generated using Phusion High-Fidelity DNA Polymerase (New England Biolabs) on a C57BL/6J genomic DNA template. The 5′ and 3′ homology arms comprising mutated exon 21 were subcloned into a parental targeting vector to achieve the positioning of the loxP and FRT sites and PGKneo cassette. For this purpose, the targeting vector was generated using bacterial artificial chromosome (BAC) clones from the C57BL/6J RPCIB-731 BAC library which then were transfected into the TaconicArtemis C57BL/6N Tac ES cell line. Homologous recombinant clones were selected using positive (PuroR) and negative (thymidine kinase–Tk) selection. The constitutive knockin allele comprising desired mutations was obtained after Flp-mediated removal of the selection marker. The targeting construct was linearized and electroporated into ES cells according to standard methods. Successful gene targeting of ES cells at the 5′ and 3′ ends was confirmed by sequencing of a ∼6 kb PCR product. Properly targeted ES cell clones were then subjected to the diploid injection into BALB/c blastocysts and implanted into foster mothers according to standard procedures. Male chimaeras resulting from the LRRK2[S910A+S935A]-targeted ES cells were bred with C57BL/6J female mice expressing Cre recombinase from the ROSA26 locus to facilitate removal of the loxP flanked PGKneo cassette *in vivo*, and germline transmission was identified by the presence of black, strain C57BL/6, offspring (G1) and PCR.

### Genotyping of mice

For LRRK2[D2017A] knockin mice, primers 5′-CCGAG-CCAAAAACTAAGCTC-3′ and 5′-CCATCTTGGGTACTT-GACC-3′ were used to detect the WT and knockin alleles (WT, 400 bp; knockin, 550 bp; heteroduplex formation). For LRRK2[S910A+S935A] knockin mice, primers 5′-GTG-CTTGAAGTTTGATCATAATGC-3′ and 5′-GCATATAGCA-TGTAGTGTCATCTCC-3′ were used to detect the WT and knockin alleles (WT, 326 bp; knockin, 401 bp; heteroduplex formation). The PCR programme consisted of 5 min at 95°C, then 35 cycles of 30 s at 95°C, 30 s at 60°C and 30 s at 72°C, and then 5 min at 72°C. DNA sequencing was used to confirm the knockin mutation and performed by DNA Sequencing & Services (MRC-PPU; http://www.dnaseq.co.uk/) using Applied Biosystems Big-Dye version 3.1 chemistry on Applied Biosystems model 3730 automated capillary DNA sequencer.

### Generation and culture of MEFs

Littermate matched WT and homozygous LRRK2[S910A+S935A] or homozygous LRRK2[R1441G] knockin MEFs were isolated from mouse embryos at embryonic day (E)12.5 resulting from crosses between heterozygous LRRK2[S910A+S935A]/WT or LRRK2[R1441G]/WT mice using a previously described protocol [34]. LRRK2[S910A+S935A] cells were genotyped as described above and LRRK2[R1441G] cells were genotyped as described previously [32]. Homozygous LRRK2[S910A+S935A] knockin as well as the WT cells generated from the same littermate were spontaneously immortalized by prolonged passaging in parallel for at least 20 passages before being used for Phos-tag experiments. Genotype of these cells was also confirmed by immunoblot analysis with anti-phospho-Ser^910^ and -Ser^935^ antibodies (Figure 8A). Homozygous LRRK2[R1441G] knockin MEFs used for the experiment shown in Figure 6(A) were used on passage 5.

Littermate matched WT and homozygous LRRK2[D2017A] knockin MEFs were isolated by Dr Francisco Inesta-Vaquera (University of Dundee) from mouse embryos at E12.5 resulting from crosses between heterozygous LRRK2[D2017A]/WT mice using a previously described protocol [34]. Cells were genotyped as described above for mice, and WT and homozygous LRRK2[D2017A] knockin cells generated from the same littermate were selected for subsequent experiments. Cells were continuously passaged in parallel for at least 20 passages before being used for Phos-tag experiments. An identical approach was used to generate littermate WT and LRRK2[S910A+S935A] and LRRK2[R1441G] knockin MEFs.

Littermate matched WT and homozygous LRRK2[G2019S]^GSK^ MEFs, littermate matched WT and homozygous LRRK2[A2016T] MEFs and littermate matched WT and homozygous LRRK2 KO MEFs were isolated as described previously and used at over passage 20 [13,35].

All MEFs were cultured in Dulbecco's modified Eagle's medium (DMEM) containing 10% FBS, 2 mM L-glutamine, 100 units/ml penicillin, 100 μg/ml streptomycin, non-essential amino acids (Life Technologies) and 1 mM sodium pyruvate (Life Technologies). All knockin and KO cell lines were verified by allelic sequencing.

### Mouse tissue lysate preparation

Frozen mouse tissues were quickly defrosted in the ice-cold lysis buffer containing 50 mM Tris/HCl, pH 7.5, 1% (v/v) Triton X-100, 1 mM EGTA, 1 mM sodium orthovanadate, 50 mM NaF, 0.1% (v/v) 2-mercaptoethanol, 10 mM 2-glycerophosphate, 5 mM sodium pyrophosphate, 0.1 μg/ml mycrocystin-LR (Enzo Life Sciences), 270 mM sucrose and Complete EDTA-free protease inhibitor cocktail (Roche) and homogenized using a POLYTRON homogenizer (KINEMATICA) on ice (5 s homogenization, 10 s interval and 5 s homogenization). Lysates were centrifuged at 20800 ***g*** for 30 min at 4°C and supernatants were used for Bradford assay and immunoblot analysis.

### Generation of Rab10 KO A549 cells

A549 cells at ∼80% confluency were co-transfected in a six-well plate with DU52110 and DU52100 plasmids using Lipofectamine LTX (Life Technologies) according to the manufacturer's instructions, with a final amount of 9 μl of Lipofectamine LTX and 2.5 μg of DNA per well. The cells were then incubated for 24 h in DMEM supplemented with 10% FBS, 2 mM L-glutamine, 100 units/ml penicillin and 100 μg/ml streptomycin. The medium was then replaced with fresh medium supplemented with 2 μg/ml puromycin. After 24 h of puromycin selection the medium was replaced again with fresh medium without puromycin and the cells were left to recover for 48 h before performing single-cell sorting. Cell sorting was performed using influx cell sorter (Becton Dickinson). Single cells were placed in individual wells of a 96-well plate containing DMEM supplemented with 10% FBS, 2 mM L-glutamine, 100 units/ml penicillin, 100 μg/ml streptomycin and 100 μg/ml Normocin (InvivoGen). After reaching ∼80% confluency individual clones were transferred into six-well plates. After reaching ∼80% confluency the clones were screened by Western blotting for the presence of Rab10. Selected clones lacking expression of Rab10 were sequenced to confirm the KO. Genomic DNA was isolated using a GenElute Mammalian Genomic DNA Miniprep Kit (Sigma–Aldrich). The PCR was performed using PfuUltra High-Fidelity DNA Polymerase (Agilent Technologies) with primers 5′-TTCCTCAAAGCTGTTCGTAGGTCG-3′ and 5′-TCCTCCCACAGGTCTTACCTATGG-3′ to amplify the region targeted for KO, followed by incubation with Taq polymerase (New England Biolabs) to add 3′ A overhangs. The PCR products were then cloned into pSC-A-amp/kan vector using StrataClone PCR Cloning Kit (Agilent Technologies). For each cloning reaction 20 positive bacterial colonies were selected and the plasmids were isolated using QIAprep Spin Miniprep Kit (Qiagen). The inserts in each individual clone were sequenced using M13 primers (DNA sequencing facility of Division of Signal Transduction Therapy at the University of Dundee). This procedure allowed us to confirm that there were no WT alleles of the Rab10 gene present in the genome of selected clone thus confirming a successful KO.

### Cell culture, transfection, treatments and lysis

HEK-293 and A549 cells were maintained in DMEM containing 10% (v/v) FBS, 2 mM L-glutamine, 100 units/ml penicillin and 100 μg/ml streptomycin at 37°C in a humidified atmosphere with 5% CO_2_. HEK-293 cells were seeded into six-well plates at 3×10^5^ cells/well, and after 24 h culture cells were transfected with Lipofectamine 2000 (Life Technologies) using 0.5 μg of the Rab10 plasmid, 2 μg of the LRRK2 plasmid and 6 μl of Lipofectamine 2000 according to the manufacturer's protocol. Cells were lysed 24 h after transfection. Inhibitors were dissolved in DMSO. An equivalent volume of DMSO was added to negative control samples. Following treatment, cells were washed with TBS (20 mM Tris/HCl, pH 7.5, and 150 mM NaCl) on ice and lysed in an ice-cold lysis buffer containing 50 mM Tris/HCl, pH 7.5, 1% (v/v) Triton X-100, 1 mM EGTA, 1 mM sodium orthovanadate, 50 mM NaF, 0.1% (v/v) 2-mercaptoethanol, 10 mM 2-glycerophosphate, 5 mM sodium pyrophosphate, 0.1 μg/ml mycrocystin-LR (Enzo Life Sciences), 270 mM sucrose and Complete EDTA-free protease inhibitor cocktail (Roche). Lysates were centrifuged at 20800 ***g*** for 15 min at 4°C and supernatants were used for Bradford assay (Thermo Scientific) and immunoblot analysis.

### Phos-tag SDS/PAGE and immunoblot analysis

Cell/tissue lysates were mixed with 4× SDS/PAGE sample buffer [250 mM Tris/HCl, pH 6.8, 8% (w/v) SDS, 40% (v/v) glycerol, 0.02% (w/v) Bromophenol Blue and 4% (v/v) 2-mercaptoethanol] and heated at 95°C for 5 min. For normal SDS/PAGE, 10–20 μg samples were loaded on to NuPAGE Bis-Tris 4–12% gels (Life Technologies) and electrophoresed at 150 V. For Phos-tag SDS/PAGE, samples were supplemented with 10 mM MnCl_2_ before loading gels. Phos-tag SDS/PAGE was carried out essentially as described previously [27]. Gels for Phos-tag SDS/PAGE consisted of a stacking gel [4% (w/v) acrylamide, 125 mM Tris/HCl, pH 6.8, 0.1% (w/v) SDS, 0.2% (v/v) *N,N,N′,N′*_tetramethylethylenediamine (TEMED) and 0.08% (w/v) ammonium persulfate (APS)] and a separating gel [12% (w/v) acrylamide, 375 mM Tris/HCl, pH 8.8, 0.1% (w/v) SDS, 75 μM Phos-tag acrylamide, 150 μM MnCl_2_, 0.1% (v/v) TEMED and 0.05% (w/v) APS]. The gel mixture was degassed for 10 min before adding TEMED and APS. After centrifugation at 20800 ***g*** for 1 min, 10–30 μg samples were loaded and electrophoresed at 70 V for the stacking part and at 150 V for the separating part with the running buffer [25 mM Tris/HCl, 192 mM glycine and 0.1% (w/v) SDS]. For Coomassie Blue staining, gels were stained with Colloidal Coomassie Blue Staining Kit (Life Technologies) according to the manufacturer's instructions. For immunoblot analysis, gels were washed for 10 min in the transfer buffer [48 mM Tris/HCl, 39 mM glycine and 20% (v/v) methanol] containing 10 mM EDTA and 0.05% (w/v) SDS three times, followed by one wash in the transfer buffer containing 0.05% SDS for 10 min. Proteins were electrophoretically transferred onto nitrocellulose membranes (Amersham Protran 0.45 μm NC; GE Healthcare) at 100 V for 180 min on ice in the transfer buffer without SDS/EDTA. Transferred membranes were blocked with 5% (w/v) non-fat dry milk (NFDM) dissolved in TBS-T [20 mM Tris/HCl, pH 7.5, 150 mM NaCl and 0.1% (v/v) Tween 20] at room temperature for 30 min. Membranes were then incubated with primary antibodies diluted in 5% NFDM and skim milk powder in TBS-T overnight at 4°C. After washing membranes in TBS-T, membranes were incubated with horseradish peroxidase-labelled secondary antibodies diluted in 5% NFDM and skim milk powder in TBS-T at room temperature for 1 h. After washing membranes in TBS-T, protein bands were detected by exposing films [Medical Film (Konica Minolta) for normal immunoblot and Amersham Hyperfilm ECL (GE Healthcare) for Phos-tag immunoblot] to the membranes using an ECL solution [Amersham ECL Western Blotting Detection Reagents (GE Healthcare) for normal immunoblot and SuperSignal West Dura Extended Duration (Thermo Fisher Scientific) for Phos-tag immunoblot].

### Purification of Rab proteins

#### Rab10

The coding sequence for human Rab10 (accession number: NM_016131.4) was cloned into pET15b so that the protein was N-terminally tagged with 6His-SUMO (clone number DU51062). BL21(DE3) cells were co-transformed with pET15b-6His-SUMO-Rab10 and a plasmid encoding the chaperone GroEL/S, and clones were allowed to grow in the presence of 100 μg/ml carbenicillin and 20 μg/ml chloramphenicol. Transformed bacteria were grown overnight and used to inoculate 6 litres of LB containing 50 μg/ml carbenicillin and 20 μg/ml chloramphenicol. After growing cells to a *D*_600_ of 0.4 at 37°C, temperature was lowered to 16°C and cells were grown until reaching a *D*_600_ of 0.6. Expression of Rab10 was induced by adding 125 μM IPTG for 14–18 h at 16°C with agitation at 200 rotations/min. Cells were collected by sedimentation and resuspended in ice-cold 50 mM Tris/HCl, pH 7.5, 250 mM NaCl, 0.2% Triton X-100, 5 mM MgCl_2_, 10 μM GDP, 1 mM tris-(2-carboxyethyl)phosphine (TCEP), 1 μM Pefabloc and 0.1% leupeptin. The suspension was sonicated and insoluble material was removed by centrifugation (20 min at 40000 ***g***). The supernatant was supplemented with 10% glycerol, 20 mM imidazole, 50 μM ATP and 1 ml Ni-agarose and incubated on a roller mixer for 1 h at 4°C. Contaminants were removed with five washes (5×12 vol.) of 50 mM Tris/HCl, pH 7.5, 250 mM NaCl, 10% glycerol, 25 mM imidazole, 5 mM MgCl_2_, 0.2% Triton X-100, 0.03% Brij-35, 10 μM GDP, 50 μM ATP and 1 mM TCEP. Rab10 was removed from the resin by cleaving the His-SUMO tag using 1 mg of a catalytic domain of SUMO-specific protease His-SENP1 (amino acids 415–643) for 16 h at 4°C and collected in four resin volumes. The protein was diluted 10-fold into 50 mM HEPES/NaOH, pH 7.5, 25 mM NaCl, 10% glycerol, 5 mM MgCl_2_, 0.03% Brij-35, 10 μM GDP, 50 μM ATP and 1 mM TCEP and purified further over a 1 ml heparin HiTrap HP column (GE Healthcare), which was developed with a total 18 ml gradient of NaCl (25–1200 mM). Rab10 was eluted in two peaks, of which the earlier peak, eluting at approximately 200 mM NaCl, contained 90% pure Rab10. The yield is very low at only 50 μg/l expression.

#### Rab8A

The coding sequence for human Rab8A (accession number: NM_005370.4) was cloned into pET15b (clone number DU47363) and purified as described previously [13].

### Assessment of kinase activity of endogenous LRRK2

In Figure 2(B), the kinase activity of endogenous LRRK2 immunoprecipitated from littermate WT and kinase-inactive LRRK2[D2017A] knockin MEFs was assessed in an *in vitro* kinase reaction as previously described [35]. Briefly, endogenous LRRK2 was immunoprecipitated from lysates (5 mg of protein) using 10 μg of anti-LRRK2 antibody UDD3 coupled to Protein A–Sepharose beads. A control was also included when UDD3 was replaced by pre-immune IgG. Peptide kinase assays were set up with immunoprecipitated LRRK2 in 50 mM Tris/HCl (pH 7.5), 0.1 mM EGTA, 10 mM MgCl_2_ and 0.1 mM [γ-^32^P]ATP (∼300–500 c.p.m./pmol, PerkinElmer) in the presence of 20 μM Nictide peptide substrate (RLGWWRFYTLRRARQGNTKQR) in the presence of either 1 μM MLi-2 or the equivalent volume of DMSO. After incubation for 20 min at 30°C with shaking, reactions were terminated by applying the reaction mixture on to P81 phosphocellulose papers and immersing in 50 mM orthophosphoric acid. After extensive washing, reaction products were quantified by Cerenkov counting. For experiments performed in Figure 7(B), the endogenous LRRK2 was immunoprecipitated from littermate WT and LRRK2[S910A+S935A] knockin MEFs as described above. Kinase assays were carried out using purified Rab8A protein as a substrate as described previously [13].

### Assessment of phosphorylation at Ser^1292^ of endogenous LRRK2

Endogenous LRRK2 was immunoprecipitated as described above from lysates (3.5 mg of protein). Immunoprecipitated LRRK2 was washed twice with the lysis buffer containing 0.5 M NaCl and eluted from the beads with 30 μl of 2× NuPAGE lithium dodecyl sulfate (LDS) Sample Buffer (Thermo Fisher Scientific). Eluted samples at 5 and 15 μl were loaded for detecting total LRRK2 and phospho-Ser^1292^ LRRK2 respectively. For detecting phospho-Ser^1292^ LRRK2 VeriBlot secondary antibody (Abcam, ab131366) was used instead of normal anti-rabbit IgG secondary antibody.

### *In vitro* phosphorylation of Rab10 by LRRK2

Purified Rab10 (6.5 μg per 25 μl reaction) was phosphorylated using full-length LRRK2[G2019S] (0.8 μg) in a buffer containing 50 mM Tris/HCl, pH 7.5, 0.1 mM EGTA, 10 mM MgCl_2_ and 1 mM ATP, in the absence or presence of the LRRK2 inhibitor MLi-2 (1 μM final concentration). A reaction where no LRRK2 was added was also included as a negative control. Assays were carried out in Dispo-Biodialysers of 1 kDa molecular mass cut-off (Sigma–Aldrich) put in 0.5 litre of the same buffer to allow for ADP exchange for the indicated times at room temperature. Kinase reactions were terminated by addition of sample buffer containing 2-mercaptoethanol.

### Isolation of B-cells from mouse spleen

Mouse B-cells were isolated from spleen using the MACSTM B-Cell Isolation Kit (Miltenyi Biotec, catalogue number 130-090-862) according to manufacturer's instructions. After isolation, B-cells were cultured in RPMI 1640 medium supplemented with 10% heat-inactivated FBS, 2 mM L-glutamine, 50 units/ml penicillin, 50 μg/ml streptomycin, sodium pyruvate and non-essential amino acids (Life Technologies) for 90 min before being treated with the LRRK2 inhibitor MLi-2 (50 nM final concentration) for 60 min.

## RESULTS

### Validation of the Phos-tag approach to assess LRRK2-mediated phosphorylation of Rab10

**Figure 1:**
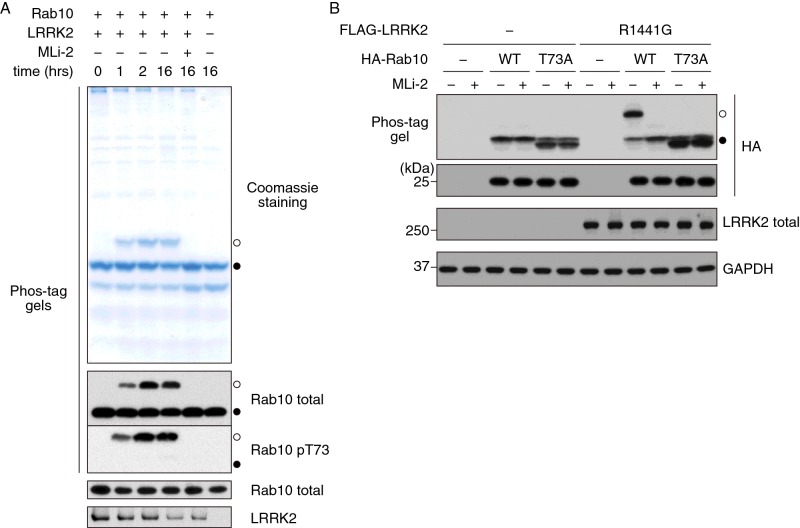
Phos-tag analysis of LRRK2 mediated Rab10 phosphorylation (**A**) Time course of LRRK2-mediated phosphorylation of recombinant Rab10, in the absence or presence of the LRRK2 inhibitor MLi-2. Rab10 phosphorylation was analysed by a Phos-tag assay using an anti-total Rab10 antibody or a phospho-specific antibody. A Coomassie Blue-stained Phos-tag gel is also shown (top panel). Control immunoblots (Rab10 total and LRRK2) were done on normal gels using the indicated antibodies (bottom panels). (**B**) HEK-293 cells were transfected with FLAG–LRRK2 R1441G and HA–Rab10 WT or T73A mutant and treated with or without 100 nM MLi-2 for 1 h. Phosphorylation of overexpressed Rab10 was analysed by a Phos-tag assay (top panel). Equal levels of expression of HA–Rab10 and FLAG–LRRK2 R1441G were confirmed by immunoblotting on normal gels using an anti-HA (second panel from the top) and anti-LRRK2 (third panel from the top) antibodies respectively. Equal loading was shown by immunoblotting with an anti-GAPDH antibody (bottom panel). Bands corresponding to phosphorylated and non-phosphorylated Rab10 were marked with open (○) and closed (●) circles respectively. Similar results were obtained in at least two separate experiments.

We first explored the effect of phosphorylation of recombinant bacterial expressed Rab10 with LRRK2[G2019S] on the electrophoretic mobility of Rab10 on Phos-tag-containing polyacrylamide gels. LRRK2 phosphorylation induced a time-dependent retardation in the migration of phosphorylated Rab10, an effect that was prevented by inclusion of the MLi-2 LRRK2 kinase inhibitor in the kinase reaction [36] (Figure 1A). Immunoblot analysis with a phospho-specific antibody confirmed that the slower migrating Rab10 species that appears following LRRK2 phosphorylation is indeed Rab10 phosphorylated at Thr^73^ (Figure 1A). We also studied LRRK2-mediated phosphorylation of HA–Rab10 following its co-expression with pathogenic LRRK2[R1441G] in HEK-293 cells. Under these conditions ∼70% of Rab10 was phosphorylated and the phosphorylation-induced mobility was blocked by mutation of the LRRK2 phosphorylation site (Thr^73^ to alanine) or by treatment of cells with MLi-2 LRRK2 inhibitor (Figure 1B).

### Use of the Phos-tag approach to assess LRRK2-mediated phosphorylation of endogenous Rab10 in MEFs

**Figure 2:**
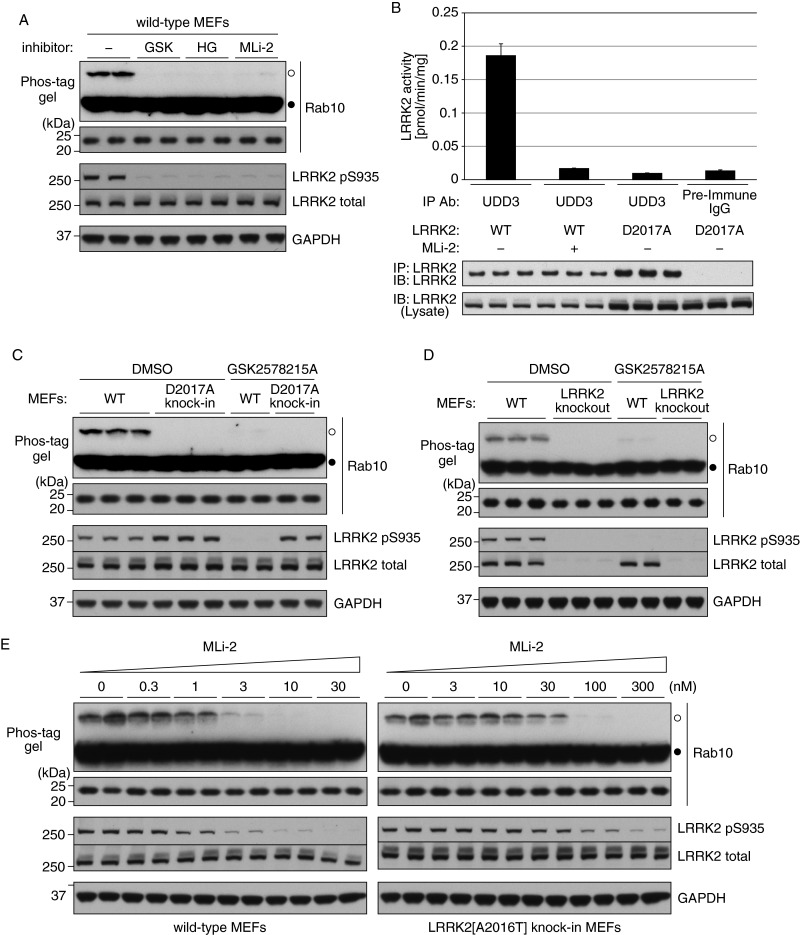
Phosphorylation of endogenous Rab10 in MEFs analysed by Phos-tag assay (**A**) WT MEFs were treated with 0.1% (v/v) DMSO (−), 1 μM GSK2578215A (GSK), 3 μM HG-10-102-01 (HG) or 10 nM MLi-2 for 1 h in duplicate. Cell lysates were prepared and Rab10 phosphorylation was analysed by a Phos-tag assay (top panel). Control immunoblots were done on normal gels with the indicated antibodies. (**B**) LRRK2 immunoprecipitated from littermate WT or kinase-inactive LRRK2[D2017A] knockin MEFs was assessed for phosphorylation of Nictide peptide substrate in the absence or presence of MLi-2 (1 μM). IgG controls were also included where LRRK2 immunoprecipitating antibody was replaced by pre-immune IgG. Western blots below show the levels of immunoprecipitated LRRK2 used for the kinase assays and LRRK2 levels in pre-immune lysates. Results are means ± S.D. (*n*=3). (**C**) Littermate WT and kinase-inactive LRRK2[D2017A] knockin MEFs were treated with or without 1 μM GSK2578215A for 1 h. Cell lysates were prepared and Rab10 phosphorylation was analysed by a Phos-tag assay (top panel). Control immunoblots were done on normal gels with the indicated antibodies. (**D**) As in (**C**) except littermate WT and LRRK2 KO MEFs were used. (**E**) Littermate WT and MLi-2-resistant LRRK2[A2016T] knockin MEFs were treated with the indicated concentrations of MLi-2 for 1 h in duplicate. Cell lysates were prepared and Rab10 phosphorylation was analysed by a Phos-tag assay (top panel). Control immunoblots were done on normal gels with the indicated antibodies. Bands corresponding to phosphorylated and non-phosphorylated Rab10 were marked with open (○) and closed (●) circles respectively. Similar results were obtained in at least two separate experiments.

We tested whether the Phos-tag approach could be used to assess LRRK2 phosphorylation of endogenous Rab10 in MEFs. The Rab10 antibody used for these studies was selective as it detected endogenous Rab10 in WT, but not in clustered regularly interspaced short palindromic repeats (CRISPR)/CRISPR-associated 9 (Cas9) Rab10 knockout A549 cells (Supplementary Figure S1). Phos-tag analysis of Rab10 derived from WT MEFs revealed that the bulk of Rab10 was in the unphosphorylated form; nevertheless, a significant minor phosphorylated Rab10 species was observed (Figure 2A). Treatment of WT MEFs with structurally diverse LRRK2 inhibitors (GSK2578215A, HG-10-102-01 and MLi-2) prevented Rab10 phosphorylation as judged by loss of the phosphorylated slower migrating Rab10 species (Figure 2A).

We next analysed Rab10 phosphorylation in MEFs derived from a novel kinase-inactive LRRK2[D2017A] knockin mouse model, described here for the first time. LRRK2 isolated from the LRRK2[D2017A] knockin MEFs is expressed at slightly elevated levels compared with that in littermate WT cells (Figure 2B). Kinase activity measurements following LRRK2 immunoprecipitation confirmed that LRRK2 in the LRRK2[D2017A] knockin cells is devoid of kinase activity (Figure 2B). Phos-tag analysis revealed that Rab10 phosphorylation was strikingly absent from LRRK2[D2017A] knockin MEFs (Figure 2C). We also observed that Rab10 was also not phosphorylated in LRRK2 knockout MEFs (Figure 2D).

**Figure 3:**
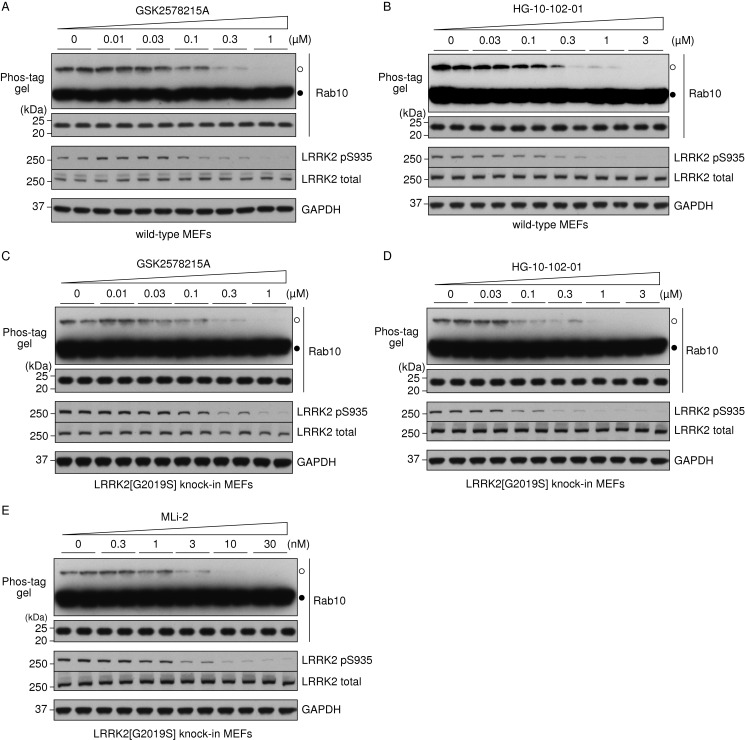
Dose-dependent inhibition of Rab10 phosphorylation in MEFs analysed by Phos-tag assay WT MEFs were treated with the indicated concentrations of (**A**) GSK2578215A or (**B**) HG-10-102-01 for 1 h in duplicate. Cell lysates were prepared and Rab10 phosphorylation was analysed by a Phos-tag assay (top panel). Control immunoblots were done on normal gels with the indicated antibodies. (**C** and **D**) As in (**A**) and (**B**) except LRRK2[G2019S]^GSK^ knockin MEFs were used. (**E**) As in (**C**) and (**D**) except MLi-2 was used. Bands corresponding to phosphorylated and non-phosphorylated Rab10 were marked with open (○) and closed (●) circles respectively. Similar results were obtained in at least two separate experiments.

Phos-tag analysis permitted detection of MLi-2 inhibition of Rab10 phosphorylation in WT and previously described MLi-2 inhibitor resistant LRRK2[A2016T] knockin MEFs [13] (Figure 2E). Doses of 3–10 nM MLi-2 suppressed Rab10 phosphorylation in WT MEFs, but concentrations of ≥100 nM were required to equivalently reduce phosphorylation in LRRK2[A2016T] knockin cells (Figure 2E). MLi-2 induced dephosphorylation of the LRRK2 Ser^935^ biomarker site [28], paralleled Rab dephosphorylation in the WT as well as the inhibitor-resistant LRRK2[A2016T] knockin MEFs (Figure 2E). Two other structurally diverse GSK2578215A [29], HG-10-102-01 [30] LRRK2 inhibitors induced a dose-dependent inhibition of Rab10 phosphorylation in WT MEFs (Figures 3A and 3B), with suppression of Rab10 phosphorylation coinciding with loss of LRRK2 Ser^935^ phosphorylation. LRRK2[G2019S]^GSK^ knockin MEFs were treated with the LRRK2 inhibitors (Figures 3C–3E), showing inhibition of Rab10 phosphorylation and loss of LRRK2 Ser^935^ phosphorylation at a similar dose to that required in WT MEFs (Figures 2E, 3A and 3B).

### LRRK2 inhibitors induce more rapid dephosphorylation of Rab10 than kinase biomarker residues (Ser^935^ and Ser^1292^)

**Figure 4:**
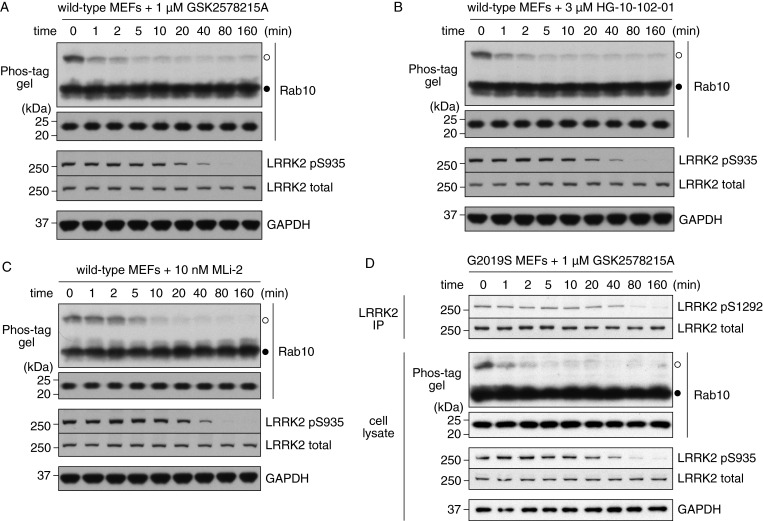
Time-course experiments to compare phosphorylation of endogenous LRRK2 Ser^935^ and endogenous Rab10 in MEFs analysed by Phos-tag assay WT MEFs were treated with (**A**) 1 μM GSK2578215A, (**B**) 3 μM HG-10-102-01 or (**C**) 10 nM MLi-2. Cell lysates were prepared at the indicated time points and Rab10 phosphorylation was analysed by a Phos-tag assay (top panel). Control immunoblots were done on normal gels with the indicated antibodies. (**D**) LRRK2[G2019S]^GSK^ knockin MEFs were treated with 1 μM GSK2578215A. Cell lysates were prepared at the indicated time points and Rab10 phosphorylation was analysed by a Phos-tag assay. Control immunoblots were done on normal gels with the indicated antibodies. Endogenous LRRK2 was also immunoprecipitated from cell lysates and blotted with the anti-pS1292 or total LRRK2 antibody (top panel). Bands corresponding to phosphorylated and non-phosphorylated Rab10 were marked with open (○) and closed (●) circles respectively. Similar results were obtained in at least two separate experiments.

**Figure 5:**
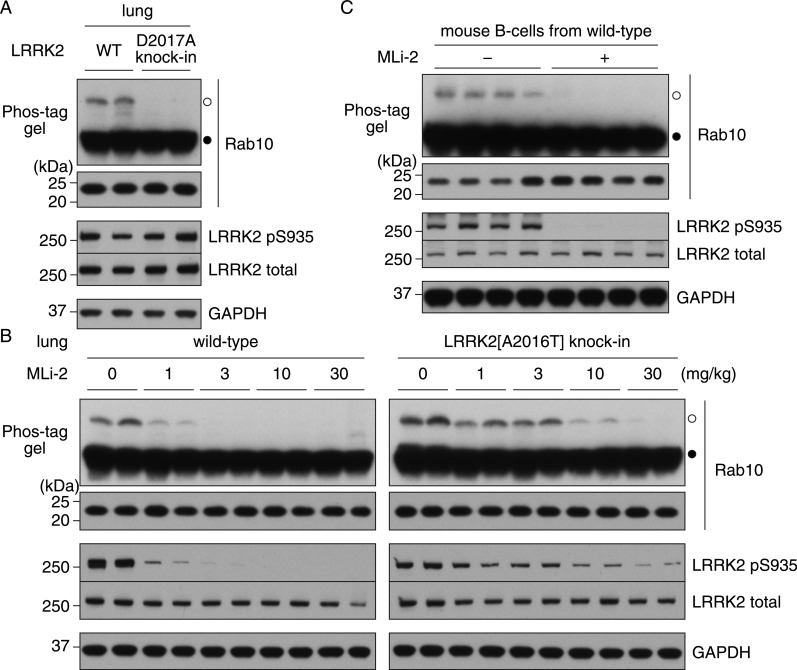
Phosphorylation of endogenous Rab10 in mouse lung and spleen-derived B-cells analysed by Phos-tag assay (**A**) Lung tissues were collected from two littermate WT and two kinase-inactive LRRK2[D2017A] knockin mice. Tissue lysates were prepared and Rab10 phosphorylation was analysed by a Phos-tag assay (top panel). Control immunoblots were done on normal gels with the indicated antibodies. (**B**) Littermate WT and MLi-2-resistant LRRK2[A2016T] mice were subcutaneously injected with the indicated doses of MLi-2 and treated for 1 h (two mice for each dose). Lung tissues were collected and Rab10 phosphorylation was analysed by a Phos-tag assay (top panel). Control immunoblots were done on normal gels with the indicated antibodies. (**C**) B-cells were isolated from eight WT mouse spleens and treated with or without 50 nM MLi-2 for 1 h (four replicates for each condition). Cell lysates were prepared and Rab10 phosphorylation was analysed by a Phos-tag assay (top panel). Control immunoblots were done on normal gels with the indicated antibodies. Bands corresponding to phosphorylated and non-phosphorylated Rab10 were marked with open (○) and closed (●) circles respectively. Similar results were obtained in at least two separate experiments.

We next compared the rate at which Rab10 and LRRK2 Ser^935^ are dephosphorylated following treatment of WT MEFs with structurally diverse kinase inhibitors. This revealed that Rab10 was rapidly dephosphorylated within 1–2 min following treatment with 1 μM GSK2578215A (Figure 4A) or 3 μM HG-10-102-01 (Figure 4B) and 5–10 min with 10 nM MLi-2 (Figure 4C). In contrast, dephosphorylation of LRRK2 Ser^935^ was markedly slower requiring 40–80 min (Figures 4A–4C). Previous work revealed that the autophosphorylation of LRRK2 at Ser^1292^ can also be deployed as a read out for LRRK2 kinase activity and that phosphorylation of this residue is enhanced by pathogenic mutations including G2019S [37]. To investigate the rate at which Ser^1292^ is dephosphorylated, we treated LRRK2[G2019S] knockin MEFs (in which Ser^1292^ is more readily detected than in WT MEFs) with 1 μM GSK2578215A for various time points. Ser^1292^ phosphorylation was analysed employing a Ser^1292^ phospho-specific antibody following immunoprecipitation of LRRK2. These studies revealed that dephosphorylation of Ser^1292^ occurred on a longer time course more similar to that of Ser^935^ requiring 80–160 min to attain maximal dephosphorylation. As observed in WT MEFs (Figure 4A), GSK2578215A induced rapid dephosphorylation of Rab10 within 1–2 min in LRRK2[G2019S] knockin MEFs (Figure 4D).

### Use of the Phos-tag approach to assess LRRK2-mediated phosphorylation of endogenous Rab10 in mouse lung and spleen derived B-cells

We next analysed Rab10 phosphorylation in littermate WT and kinase-inactive LRRK2[D2017A] knockin mouse lung tissue. This revealed that phosphorylation of Rab10 was readily observed in WT but not in the kinase-inactive LRRK2[D2017A] knockin lung (Figure 5A). Injection of WT mice with doses of 1–3 mg/kg MLi-2 blocked Rab10 phosphorylation, whereas doses of ≥10 mg/kg MLi-2 were needed to induce equivalent blockade in LRRK2[A2016T] inhibitor-resistant lung (Figure 5B). MLi-2 induced dephosphorylation of LRRK2 Ser^935^ paralleled Rab dephosphorylation, with significantly higher doses of MLi-2 required to induce equivalent Ser^935^ and Rab10 dephosphorylation in LRRK2[A2016T] lung compared with WT (Figure 5B). Phosphorylated Rab10 was also detected in splenic B-cells derived from WT mice, which was lost following incubation of B-cells with MLi-2 in RPMI 1640 medium for 60 min prior to cell lysis (Figure 5C).

### Use of the Phos-tag approach to assess the impact of LRRK2 pathogenic mutations

**Figure 6:**
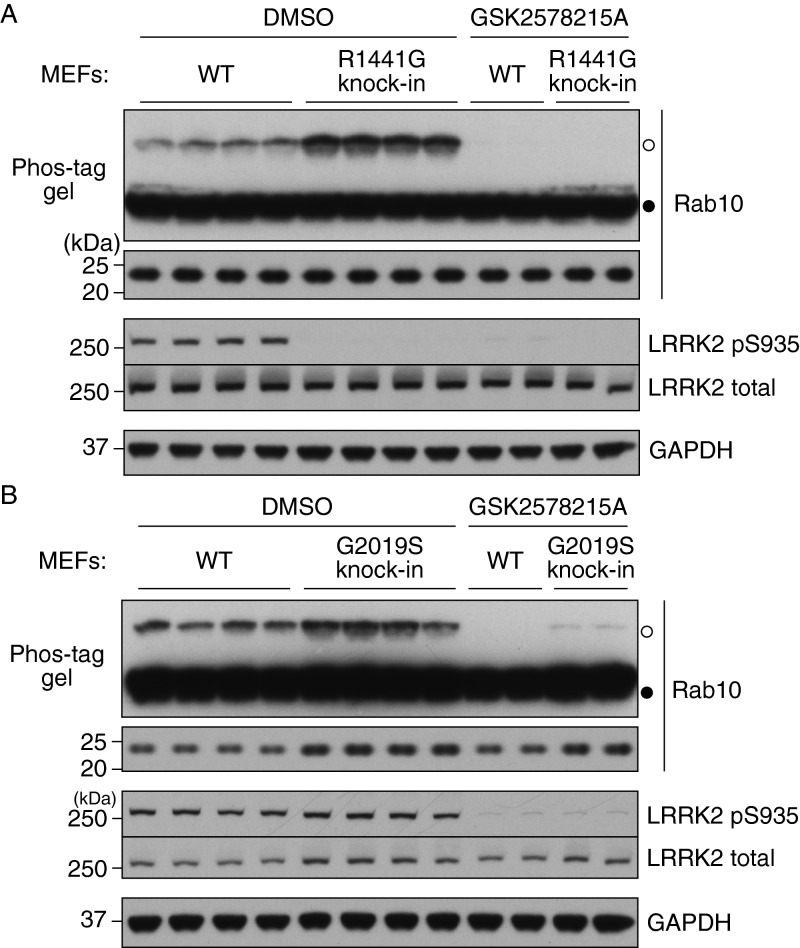
Phosphorylation of endogenous Rab10 in MEFs harbouring pathogenic mutations analysed by Phos-tag assay (**A**) Littermate WT and pathogenic LRRK2[R1441G] knockin MEFs were treated with or without 1 μM GSK2578215A for 1 h. Cell lysates were prepared and Rab10 phosphorylation was analysed by a Phos-tag assay (top panel). Control immunoblots were done on normal gels with the indicated antibodies. (**B**) As in (**A**) except littermate WT and pathogenic LRRK2[G2019S]^GSK^ knockin MEFs were used. Bands corresponding to phosphorylated and non-phosphorylated Rab10 were marked with open (○) and closed (●) circles respectively. Similar results were obtained in at least two separate experiments.

We next employed the Phos-tag approach to assess the impact of homozygous LRRK2[R1441G] (Figure 6A) and LRRK2[G2019S]^GSK^ (Figure 6B) knockin mutations on LRRK2 Rab10 phosphorylation in MEFs. Compared with WT controls, the LRRK2[R1441G] knockin enhanced Rab10 phosphorylation ∼3–4-fold (Figure 6A) and the G2019S mutation enhanced phosphorylation ∼2-fold (Figure 6B). In both R1441G and G2019S knockin MEFs, LRRK2 inhibitors suppressed Rab10 phosphorylation (Figure 6). Consistent with a previous report [12], the R1441G knockin mutation markedly inhibited basal levels of LRRK2 Ser^935^ phosphorylation (Figure 6B).

**Figure 7:**
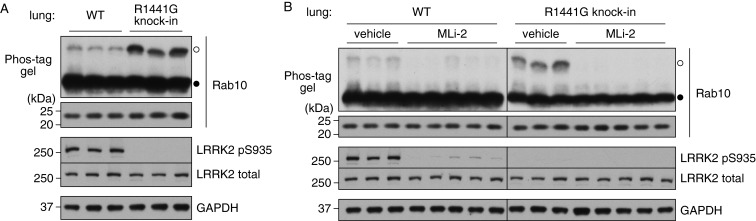
Phosphorylation of endogenous Rab10 in mouse lung harbouring pathogenic mutations analysed by Phos-tag assay Littermate WT and pathogenic LRRK2[R1441G] knockin mice were subcutaneously injected with vehicle only or MLi-2 at 3 mg/kg and treated for 1 h (three mice for vehicle control and five mice for MLi-2). Lung tissues were collected and Rab10 phosphorylation was analysed by a Phos-tag assay (top panel). Control immunoblots were done on normal gels with the indicated antibodies. (**A**) Side-by-side comparison of WT and LRRK2[R1441G] knockin lungs on a same gel. (**B**) Side-by-side comparison of lungs injected with vehicle or MLi-2 on a same gel. Bands corresponding to phosphorylated and non-phosphorylated Rab10 were marked with open (○) and closed (●) circles respectively. Similar results were obtained in at least two separate experiments.

We next analysed Rab10 phosphorylation in littermate WT and LRRK2[R1441G] knockin mouse lung tissue. This revealed that phosphorylation of Rab10 was markedly elevated in LRRK2[R1441G] knockin lung compared with WT (Figure 7A). Injection of 3 mg/kg MLi-2 for 60 min blocked Rab10 phosphorylation in both WT and LRRK2[R1441G] knockin mouse lung (Figure 7B).

### Use of the Phos-tag approach to assess the impact of S910A/S935A mutations

**Figure 8:**
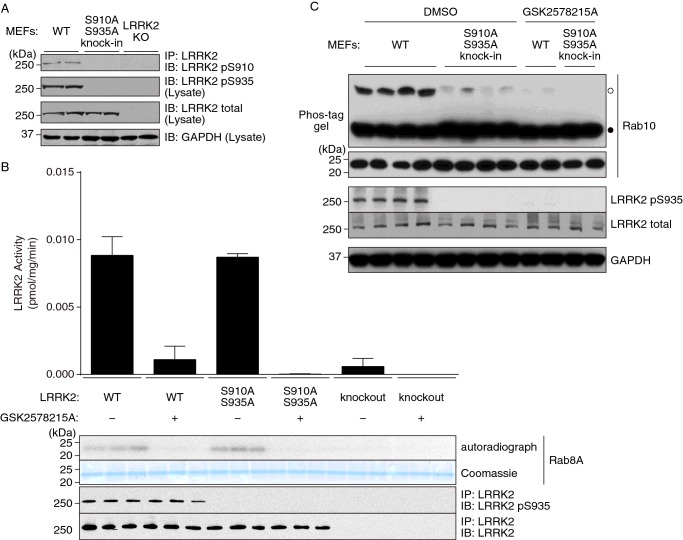
Phosphorylation of endogenous Rab10 in MEFs harbouring S910A/S935A knockin mutation analysed by Phos-tag assay (**A**) Phosphorylation of LRRK2 at Ser^910^ was analysed by immunoprecipitating LRRK2 from cell lysates of WT, LRRK2[S910A+S935A] knockin and LRRK2 KO MEFs and immunoblotting with an anti-pSer^910^ antibody (top panel). Phosphorylation of LRRK2 at Ser^935^ was analysed by immunoblotting of the cell lysates with an anti-pSer^935^ antibody (second panel from the top). Equal expression of LRRK2 in WT and knockin MEFs lysates was confirmed by immunoblotting with an anti-total LRRK2 antibody (third panel from the top). (**B**) Endogenous LRRK2 proteins were immunoprecipitated with a monoclonal anti-total LRRK2 antibody from WT, LRRK2[S910A+S935A] knockin and LRRK2 KO MEFs. Purified LRRK2 proteins were assessed for phosphorylation of Rab8A protein in the absence or presence of GSK2578215A. Immunoprecipitates were then subjected to electrophoresis on a polyacrylamide gel, autoradiography and immunoblot analysis with the indicated antibodies. After autoradiography, the bands corresponding to Rab8A were cut out to measure the radioactivity by scintillation counting. Results are means ± S.D. (*n*=3). (**C**) Littermate WT and LRRK2[S910A+S935A] knockin MEFs were treated with or without 1 μM GSK2578215A for 1 h. Cell lysates were prepared and Rab10 phosphorylation was analysed by a Phos-tag assay (top panel). Control immunoblots were done on normal gels with the indicated antibodies. Bands corresponding to phosphorylated and non-phosphorylated Rab10 were marked with open (○) and closed (●) circles respectively. Similar results were obtained in at least two separate experiments.

There is significant interest in understanding the roles that LRRK2 phosphorylation at LRRK2 Ser^910^ and Ser^935^ residues plays in controlling LRRK2 activity, as these phosphorylations regulate interaction of LRRK2 with 14-3-3 proteins and are also sensitive to LRRK2 inhibitors [12,28]. To better understand the role of Ser^910^ and Ser^935^ phosphorylations, we generated homozygous LRRK2[S910A+S935A] knockin MEFs. Immunoblot analysis confirmed that the LRRK2[S910A+S935A] mutant kinase was expressed at the same level as LRRK2 derived from littermate WT cells (Figure 8A). Moreover, following immunoprecipitation, the LRRK2[S910A+S935A] mutant was capable of phosphorylating recombinant Rab8A *in vitro* to a similar extent as the WT LRRK2 (Figure 8B). Rab8A rather than Rab10 was used for these experiments as rates of phosphorylation of Rab8A by immunoprecipitated endogenous LRRK2 was much higher and could be more robustly quantified than with Rab10. Strikingly, we observed that endogenous Rab10 phosphorylation was markedly reduced in the LRRK2[S910A+S935A] knockin MEFs compared with littermate-derived WT cells (Figure 8C).

## DISCUSSION

Here we show that the Rab10 Phos-tag assay can readily be used to assess LRRK2-mediated phosphorylation of endogenous Rab10 in MEFs, mouse lung, mouse spleen-derived B-cells. We expect that the Rab10 Phos-tag assay will work in other cell lines in which LRRK2 and Rab10 are well expressed. The Rab10 Phos-tag assay is straightforward, necessitating only SDS/polyacrylamide gel and immunoblotting apparatus and moderate amounts of cell extracts (10–45 μg of protein). Moreover, the two key reagents required for the assay, namely the anti-Rab10 monoclonal antibody and Phos-tag acrylamide are both commercially available. To reduce assay costs, we undertook chemical synthesis of the Phos-tag acrylamide reagent. It should also be noted that Phos-tag reagent requires Mn^2+^ ions in order to interact with phosphate groups [25–27]. We have also found that the Phos-tag acrylamide reagent can undergo polymerization following long-term storage which results in reduced separation of dephosphorylated and LRRK2-phosphorylated Rab10. The optimal conditions we have found to store Phos-tag acrylamide is 5 mM in aqueous solution at 4°C in black tubes that block out light as the reagent is light-sensitive. We would also recommend that purity of Phos-tag acrylamide be assessed by HPLC analysis periodically. We would also recommend that if samples to be analysed and/or the SDS sample buffer contain EDTA, an excess of MnCl_2_ over EDTA is added to the sample prior to loading the samples on to the Phos-tag gel. A single researcher could readily analyse a few dozen of samples per day using the Rab10 Phos-tag assay.

The finding that diverse LRRK2 inhibitors, kinase-inactivating LRRK2[D2017A] knockin mutation as well as LRRK2 knockout, ablate all detectable phosphorylation of Rab10, strongly suggests that LRRK2 is the major kinase that phosphorylates Rab10 at least in MEFs and lung tissue that we have analysed. The finding that LRRK2[A2016T] inhibitor-resistant knockin increases the dose of LRRK2 inhibitor required to reduced Rab10 phosphorylation in both MEFs and mouse lung provides a fundamental demonstration that LRRK2 is the major kinase controlling Rab10 phosphorylation in MEFs.

Another advantage of the Phos-tag method is that it allows assessment of stoichiometry of phosphorylation. In MEFs and lung tissue that we have analysed, the data indicate that only a small fraction of Rab10 is phosphorylated at steady state. This probably accounts for why it was challenging to identify phosphorylated species of Rab10 by mass spectrometry, as such a low proportion of the Rab protein is phosphorylated by LRRK2 *in vivo*. However, the low basal levels of LRRK2-phosphorylated Rab10 may make it easier to monitor the impact of activating LRRK2 pathogenic mutations have on enhancing Rab10 phosphorylation (Figure 6 and 7). We have also examined total brain and kidney tissue extracts to see whether we could detect LRRK2-mediated phosphorylation of Rab10, but failed to observe significant Rab10 phosphorylation using the described Phos-tag assays under conditions where LRRK2 phosphorylation of Rab10 in lung and spleen was observed. Further work is warranted to develop methodology to assess LRRK2 phosphorylation of Rab10 in brain and kidney.

In the future it will be interesting to explore whether it is possible to observe LRRK2-dependent phosphorylation of Rab proteins using the Phos-tag approach in human derived cells such as fibroblast, peripheral blood mononuclear cells (PBMCs) or other blood cells, as well as bodily fluids such as in cerebrospinal fluid. It will also be important to explore whether elevated Rab protein phosphorylation can be observed in Parkinson's disease patients who are carriers of LRRK2 mutations and whether a subgroup of Parkinson's disease patients with idiopathic disease also display elevated Rab phosphorylation. For the benefit of future clinical trials of LRRK2 inhibitors, it would be desirable to determine whether target engagement of LRRK2 inhibitors could be demonstrated by monitoring the effect these compounds have on Rab protein phosphorylation in human blood cells. It will also be intriguing to investigate whether the Rab Phos-tag assay can be used to detect LRRK2-phosphorylated Rab proteins in human urinary exosomes that contain LRRK2 [38]. Recent studies have reported elevated phosphorylation of LRRK2 at its Ser^1292^ autophosphorylation site [37] in urine exosomes and concluded that this can predict Parkinsonian phenotypes in G2019S LRRK2 subjects [39].

There has been a lot of interest in studying the roles of the LRRK2 Ser^910^ and Ser^935^ phosphorylation sites, as these mediate 14-3-3 binding and become dephosphorylated when cells are exposed to LRRK2 inhibitors [12,28]. Most of the data suggest that Ser^910^ and Ser^935^ are likely to be phosphorylated by kinases distinct to LRRK2 [12,28]. Although several candidates for the LRRK2 Ser^910^ and Ser^935^ kinase(s) have been proposed [40–42], further studies are required to pinpoint these kinase(s) and characterize how inhibition of LRRK2 leads to dephosphorylation of these residues. Consistent with the notion that an LRRK2-distinct kinase phosphorylates Ser^935^, we observe that Ser^935^ is still phosphorylated in the LRRK2[D2017A] kinase-inactive MEFs (Figure 2C) and lungs (Figure 5A). However, following MLi-2 administration, in contrast with wild type situation where Ser^935^ becomes dephosphorylated, in the LRRK2[D2017A] knockin MEFs, Ser^935^ is not dephosphorylated (Figure 2C). This is consistent with a model in which the LRRK2 Ser^935^ kinase is uncoupled from LRRK2 in the LRRK2[D2017A] knockin MEFs. The finding that treatment of cells with LRRK2 inhibitors induces more rapid dephosphorylation of Rab10 (1–2 min with GSK2578215A and HG-10-102-01) than Ser^935^ (40–80 min, Figures 4A–4C), is consistent with the regulation of Rab10 being directly mediated by LRRK2, whereas phosphorylation of Ser^935^ is indirectly controlled. The rapid dephosphorylation Rab10 that is observed following suppression of LRRK2 kinase activity may indicate that the phosphatase that acts on Rab10 is highly active and/or the Thr^73^ residue is exposed and accessible to the phosphatase. In contrast, dephosphorylation of the Ser^1292^ autophosphorylation site of LRRK2 was significantly slower than Rab10, necessitating 40–80 min (Figure 4D). This slower dephosphorylation might result if the phosphatase that targets Ser^1292^ had low activity and/or access of phosphorylated Ser^1292^ to the protein phosphatase was hindered.

The finding that the LRRK2[S910A+S935A] knockin mutation suppresses phosphorylation of Rab10 in MEFs, provides evidence for a functional role of Ser^910^ and Ser^935^ phosphorylation in enabling LRRK2 to optimally phosphorylate Rab GTPases. More work is needed to unravel this mechanism. One possibility is that this is mediated through localization of LRRK2. Previous work in a HEK-293 cell overexpression system suggested that the LRRK2[S910A+S935A] mutant was assembled into inclusion-like bodies very different from the WT LRRK2 that was diffusely localized throughout the cytosol. As functional Rab proteins are largely localized on membranes, perhaps LRRK2 Ser^910^ and Ser^935^ phosphorylation and 14-3-3 binding facilitate recruitment of LRRK2 on to membranes where it can phosphorylate Rab proteins.

## Abbreviations

APS: ammonium persulfate
BAC: bacterial artificial chromosome
Cas9: CRISPR-associated 9
COR: C-terminal of ROC (Ras of complex GTPase domain)
CRISPR: clustered regularly interspaced short palindromic repeats
DMEM: Dulbecco's modified Eagle's medium
E: embryonic day
ES: embryonic stem
GAPDH: glyceraldehyde-3-phosphate dehydrogenase
HA: haemagglutinin
HEK: human embryonic kidney
KO: knockout
LRRK2: leucine-rich repeat kinase 2
MEF: mouse embryonic fibroblast
NFDM: non-fat dry milk
TCEP: tris-(2-carboxyethyl)phosphine
TEMED: *N,N,N′,N′*_tetramethylethylenediamine
WT: wild-type
